# Lateral Semicircular Canal Asymmetry in Idiopathic Scoliosis: An Early Link between Biomechanical, Hormonal and Neurosensory Theories?

**DOI:** 10.1371/journal.pone.0131120

**Published:** 2015-07-17

**Authors:** Martin Hitier, Michèle Hamon, Pierre Denise, Julien Lacoudre, Marie-Aude Thenint, Jean-François Mallet, Sylvain Moreau, Gaëlle Quarck

**Affiliations:** 1 Department of Otolaryngology—Head and Neck Surgery, CHU de Caen, Caen, F-14000, France; 2 Department of Anatomy, UNICAEN, Caen, 14032, France; 3 Department of Pharmacology and Toxicology; School of Medical Sciences and Brain Health Research Center, University of Otago, Dunedin, New Zealand; 4 U 1075 COMETE, INSERM, Caen, 14032, France; 5 Department of Neuroradiology, CHU de Caen, Caen, 14000, France; 6 Department of Paediatric surgery, CHU de Caen, Caen, 14000, France; Tokai University, JAPAN

## Abstract

**Introduction:**

Despite its high incidence and severe morbidity, the physiopathogenesis of adolescent idiopathic scoliosis (AIS) is still unknown. Here, we looked for early anomalies in AIS which are likely to be the cause of spinal deformity and could also be targeted by early treatments. We focused on the vestibular system, which is suspected of acting in AIS pathogenesis and which exhibits an end organ with size and shape fixed before birth. We hypothesize that, in adolescents with idiopathic scoliosis, vestibular morphological anomalies were already present at birth and could possibly have caused other abnormalities.

**Materials and Methods:**

The vestibular organ of 18 adolescents with AIS and 9 controls were evaluated with MRI in a prospective case controlled study. We studied lateral semicircular canal orientation and the three semicircular canal positions relative to the midline. Lateral semicircular canal function was also evaluated by vestibulonystagmography after bithermal caloric stimulation.

**Results:**

The left lateral semicircular canal was more vertical and further from the midline in AIS (*p* = 0.01) and these two parameters were highly correlated (*r* = -0.6; *p* = 0.02). These morphological anomalies were associated with functional anomalies in AIS (lower excitability, higher canal paresis), but were not significantly different from controls (*p*>0.05).

**Conclusion:**

Adolescents with idiopathic scoliosis exhibit morphological vestibular asymmetry, probably determined well before birth. Since the vestibular system influences the vestibulospinal pathway, the hypothalamus, and the cerebellum, this indicates that the vestibular system is a possible cause of later morphological, hormonal and neurosensory anomalies observed in AIS. Moreover, the simple lateral SCC MRI measurement demonstrated here could be used for early detection of AIS, selection of children for close follow-up, and initiation of preventive treatment before spinal deformity occurs.

## Introduction

Adolescent idiopathic scoliosis (AIS) is characterized by a spinal deformity of unknown origin and affects 3% of children between the ages of 10 and 16 worldwide[1–4] [1,2], resulting in pain, poor self image with social consequences, and the possible burden of heavy treatment with a brace or spine surgery[3,5]. Even if the level of proof is currently weack[6], the spinal deformity is often associated with other morphological (e.g. ribs, pelvis, arms, skull)[7–10], hormonal (e.g. leptin, melatonin signaling) [11–13], and neurosensory anomalies (e.g. vestibular, neurosensory integration)[14–17]. Authors have identified genes[18–23] and suspected environmental factors [24,25], and have elaborated theories to link these factors to the anomalies (for review see [26–28]). However, the pathogenesis of AIS remains unknown and is probably multifactorial.[29]

One main difficulty is that some anomalies could be either the cause or the consequence of others. For example, the spinal deformity could be the cause or the consequence of limb asymmetry, or both could be the result of a common cause [27]. Spine deformity could also be the consequence of brain anomalies [30,31] or its causes, with the brain trying to compensate for the postural instability [32].

One strategy to be used to clarify the pathogenesis would be to establish the chronology of AIS anomalies. This could help to identify causes of AIS, which should be those that appear earliest. Ultimately, this chronology may help to design new treatments early enough to stop the disease at the earliest stage.

Here, we propose establishing a chronologic landmark in the vestibular system. Several studies argue for a vestibular impairment in AIS [14,15,33]. Additionally, vestibular lesion in guinea pigs or Xenopus have reproduced scoliosis, which indicates that the vestibule is a possible cause of AIS [34,35]. The mechanism of vestibular induced scoliosis is more likely an asymmetry of the vestibulospinal pathway leading to an imbalance in paraspinal muscles. Most notably, the size and shape of the vestibular organ are fixed by the ossification of the otic capsule before birth, much as a fossil in rock [36–43]. Therefore, the shape of the adolescent vestibule (i.e. bony labyrinth) is thought to be similar to the shape exhibited at birth. We thus propose studying the AIS labyrinth to determine early malformation. More precisely, we focused on the lateral semicircular canal (SCC) which can be both visualized by MRI and examined by a caloric test, allowing the evaluation of the right and left sides independently [44]. The lateral SCC is most frequently affected by malformation in the general population, probably because it is the last to be formed and ossified [45]. We hypothesized that in AIS, the lateral SCC could present early orientation troubles associated with functional impairment.

## Patients and Method

**Fig 1:**
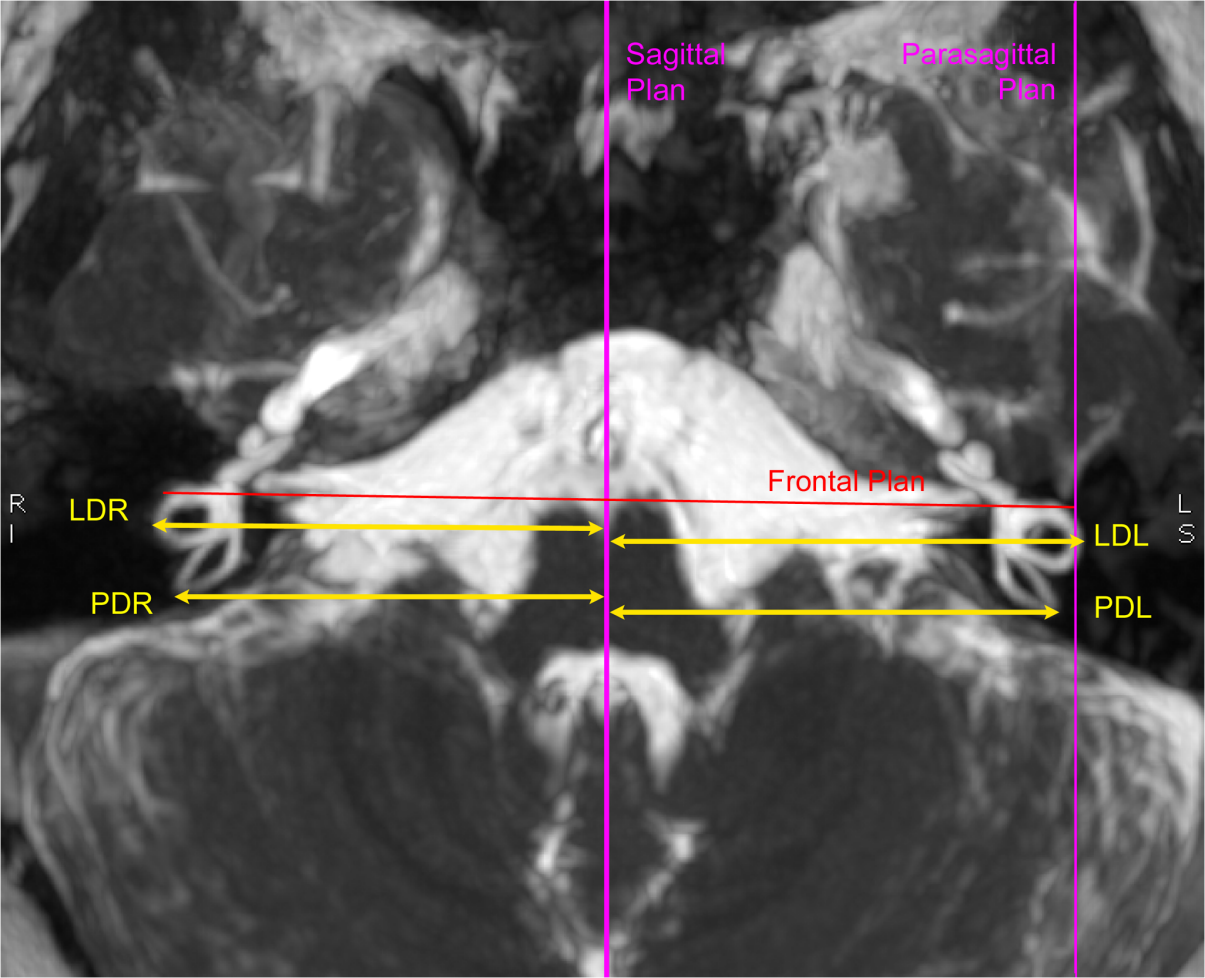
Definition of planes and semicircular canal position measurements. T2 3D MRI showing sagittal, parasagittal, and frontal planes. Distances of the semicircular canal are defined as: “*lateral distance right*” (LDR) for the right lateral canal, “*lateral distance left*” (LDL) for the left lateral canal, “*posterior distance right*” for the right posterior canal (PDR), and “*posterior distance left*” (PDL) for the left posterior canal.

**Fig 2:**
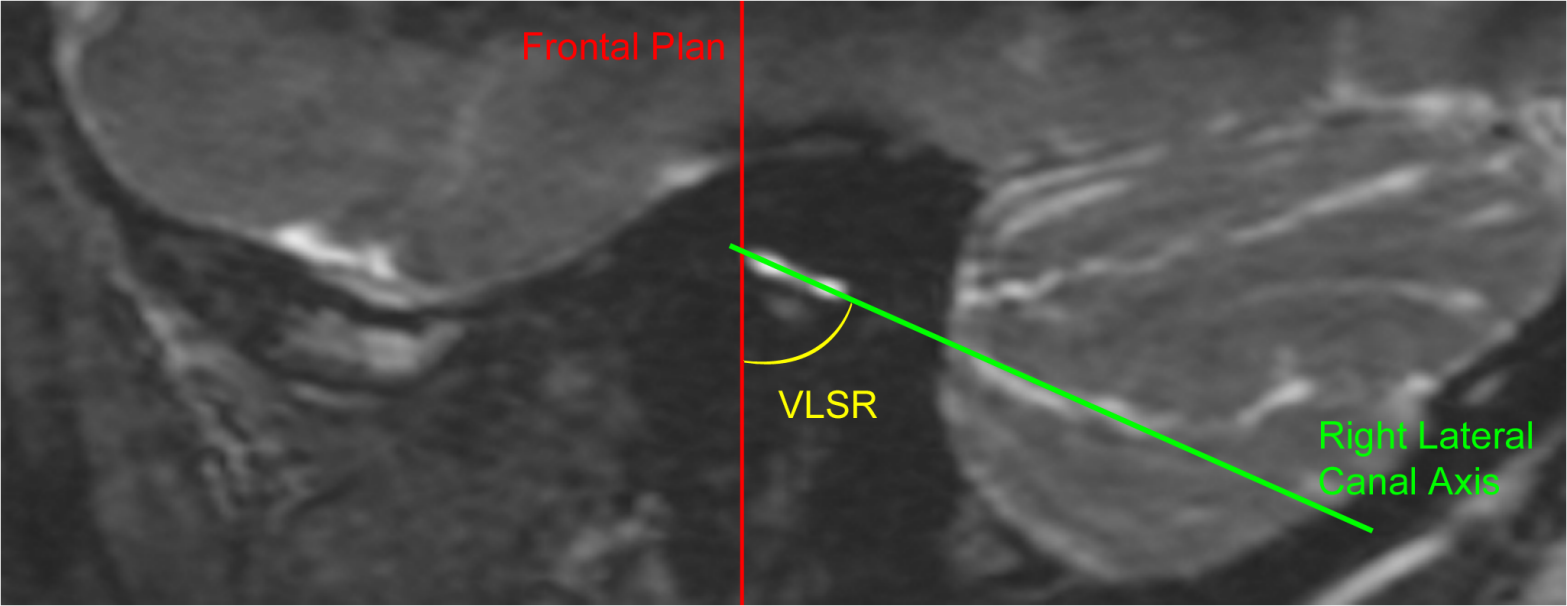
Measurement of lateral semicircular canal orientation in the parasagittal plane. T2 MRI image in the parasagittal plane showing the **“** *Vertical Lateral canal Sagittal angle Right*” (VLSR) formed between the right lateral semicircular canal and the vertical (represented by the frontal plane).

**Fig 3:**
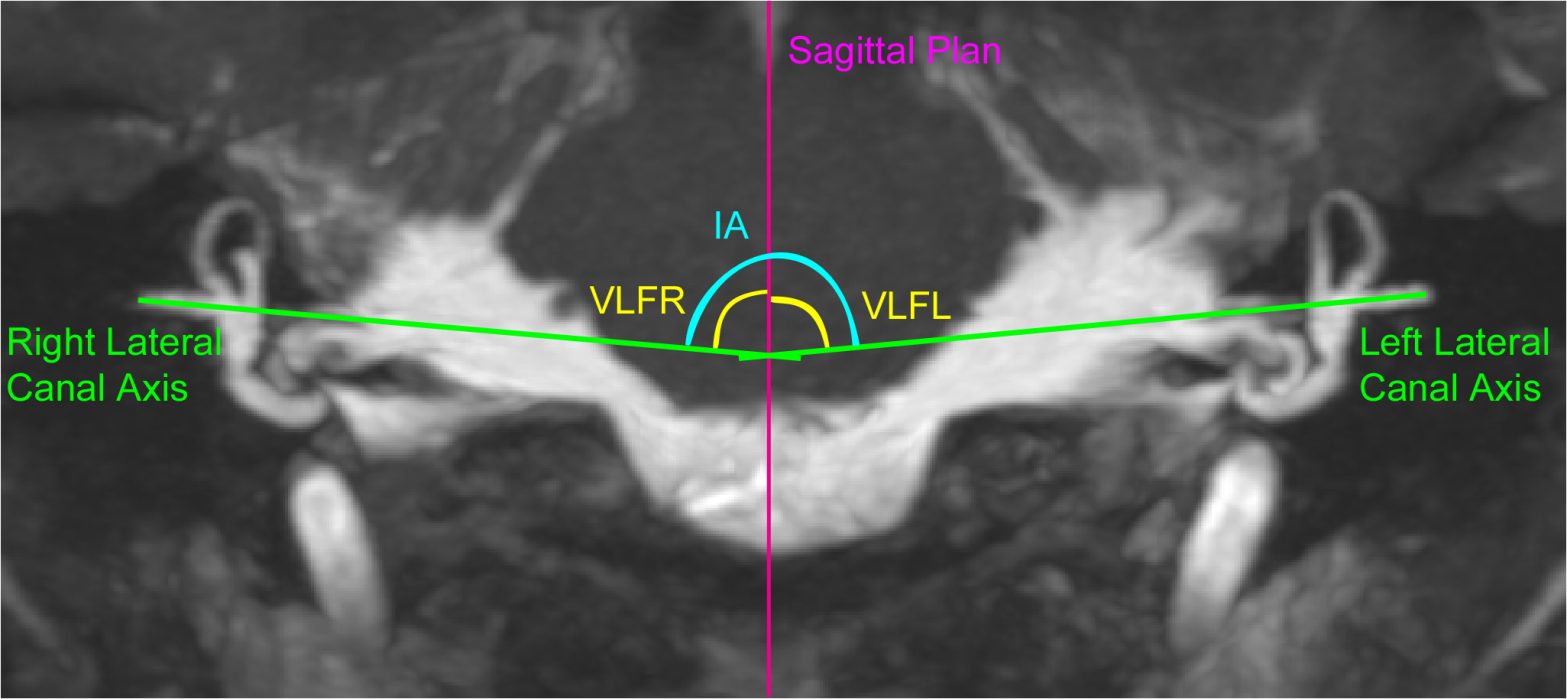
Measurement of the lateral semicircular canal in the frontal plane. MRI image in the frontal plane showing the “*Intercanal Angle*”(IA) formed by the axis of the right and left lateral canals; the “*Vertical Lateral canal Frontal angle Right*”(VLFR) formed between the right lateral SCC and the vertical axis (defined by the sagittal plane); and the” *Vertical Lateral canal Frontal angle Left” (*VLFL) formed between the left lateral SCC and the vertical axis.

We conducted a prospective case-control study including 18 cases of idiopathic scoliosis and 9 controls approved by the Nord Ouest III Ethics Committee (approval no. 2008 A00598-47). Because all cases and controls were minor, informed written consents were obtained from their parents, caretakers or guardians. One individual gave written informed consent (as outlined in PLoS consent form) to publish the image in Fig 1, Fig 2 and Fig 3 of this manuscript.

### Participants

Inclusion criteria for the scoliosis group included females and males 10–18 years of age presenting untreated idiopathic scoliosis with a Cobb angle between 10 and 50°. The types of scoliosis were determined according to the definition of the Scoliosis Research Society: the maximum convexity (i.e. apex) gives the side (right or left) and the location of the scoliosis. An apex located between T2 vertebra and T11-T12 disc defined thoracic scoliosis, between T12 and L1 thoracolumbar scoliosis and between L1-L2 discs and L4 lumbar scoliosis[46]. Controls consisted of adolescents recruited in the same school environment as scoliosis participants, but with no particular medical history. Exclusion criteria for both groups were: non-idiopathic scoliosis, medical history of orthopedic, endocrine, neurologic, or ear disease. Each participant (scoliosis or control) was assessed by an orthopedic surgeon (JFM), with evaluation of the scoliosis location, orientation, and severity (i.e. Cobb angle on X-ray). An ENT specialist (JL) also performed an otoscopy, a tympanometry, and tonal audiometry exams.

### Radioanatomic measurements

We used 1.5T MRI (General Electric) as a non irradiating imaging technique, with a T2 3D FastSpin Echo acquisition (scan time = 3 500 ms; echo time = 110 ms; and slice thickness = 0.5 mm). We have used a field of view (i.e. FOV) measuring 180 mm X 180 mm with a slice thickness of 0.6 mm and a matrix of 288 X 288. The resulting voxel sized 0.6 X 0.6 x 0.6 mm^3^. Further interpolation allows one to reduce the voxel to 0.3 X 0.3 X 0.6 mm^3^

Orientation of the lateral SCC in the parasagittal and the frontal plane was analyzed by two different neuroradiologists blinded to one another and to the study group.

### Plane definition

The sagittal plane was defined according to fixed neuroanatomic median landmarks (e.g. the mesencephalic aqueduct, the 4^th^ ventricle, the anterior median fissure). The right and left parasagittal planes are the 2 planes parallel to the sagittal plane running through the right and left lateral SCC, respectively. The frontal plane is defined as perpendicular to the sagittal and parasagittal planes (**Fig 1**).

### Analysis in the parasagittal plane

We evaluated the orientation of the lateral SCC in the parasagittal plane compared to the vertical axis. The vertical axis was defined as the frontal plane running through the most anterior part of the lateral SCC. We called the angle formed between the vertical and the lateral SCC the Vertical Lateral canal Sagittal angle: VLSR for the right canal and VLSL for the left (**Fig 2**).

### Analysis in the frontal plane

We evaluated the orientation of the lateral SCC in the frontal plane by means of both its angle with the vertical axis and the angle between the 2 lateral SCC: the angle between the lateral SCC and the vertical axis (defined by the sagittal plane) was called the Vertical Lateral canal Frontal angle: VLFR for the right SCC, and VLFL for the left. The angle between the right lateral SCC and the left lateral SCC was called the Intercanal Angle (IA) (**Fig 3).**

### Position of the three semi circular canals

We assessed the position of the most lateral point of the lateral SCC (i.e. LCSl defined by [42]) from the sagittal plane. We called this measure the Lateral Distance: LDR for the right lateral SCC, and LDL for the left SCC.

Similarly, the distance of the most posterior part of the posterior SCC from the sagittal plane was called the Posterior Distance: PDR for the right posterior SCC and PDL for the left. The the distance of the most superior part of the anterior SCC was called the *Anterior Distance*: *ADR* for the right anterior SCC, and *ADL* for the left. (**Fig 1).**

### Morphologic asymmetry

We evaluated the asymmetry between the right and left vestibule by comparing the difference between measures from the right side and measures from the left side (e.g. VLFR-VLFL). This analysis takes into account the side of the asymmetry. Right asymmetry will result in a positive value and left asymmetry results in negative values. Additionally, we evaluated the absolute asymmetry, which is the asymmetry regardless of the side. The absolute asymmetry of a morphologic parameter is calculated by the absolute difference between the right and the left parameters (e.g. [VLFR-VLFL]).

### Caloric test

The function of the lateral SCC was evaluated by vestibulonystagmography after alternate binaural bithermal caloric stimulation (250 mL warm water at 44°C for 30 s followed by 250 mL cold water at 30°C for 30 s) [47]. We used a VNG Ulmer-Synapsis device to evaluate the vestibular excitability, the directional preponderance, and the canal paresis index.

Among these three parameters, the canal paresis index is the only one which compares the function of the right and left lateral SCC (normal<15%) [48–50]. Additionally, the directional preponderance indicates stronger nystagmus beats in one side, independently of which lateral SCC is stimulated (normal<2°/s) [48–50].

### Types of comparison

We compared the morphologic parameters (angles or distances) and the functional parameters between scoliosis and control groups.

We also observed the scoliosis group for associations between the side of the scoliosis and asymmetry of morphologic or functional vestibular parameters. We then searched for correlations between the degree of scoliosis (i.e. Cobb angle) and morphologic (i.e. orientation and position) or functional (i.e. caloric test) vestibular parameters.

Finally, we studied relations between morphologic and functional vestibular parameters.

### Statistical analysis

Comparison between sex ratio and age in scoliosis and control groups were tested by Pearson’s chi-square test and a *t*-test, respectively. Both radiologists' measurements were compared using a *t*-test to check for non significant differences. Then, analysis was done using the means of the two radiologists' measurements. Outliers were identified using the Hoaglin method [51]. We compared scoliosis and control groups for morphologic and functional vestibular parameters with a *t*-test or a Mann-Whitney U test depending on the distribution of each parameter. Effect size was calculated with the Pearson product-moment correlation coefficient (*r*) after the *t*-test, or as: r = Z / √N, after the Mann-Whitney U test. We used Fisher's exact test to compare the normality of vestibular function because there was only 1 case (i.e.fewer than 5 cases) of abnormal vestibular function in the control group. We used one-way ANOVA to compare the 3 types of scoliosis location for morphologic and functional vestibular parameters. The same method was used for the 3 types of scoliosis lateralization (right scoliosis, left scoliosis, double side scoliosis). Comparison combining both scoliosis location and side was done by two-ways ANOVA. Comparison between side of scoliosis and side of morphologic or functional asymmetry was assessed with Pearson’s chi-square test. We analyzed correlations between vestibular morphologic parameters and Cobb angle, or functional vestibular parameters using Pearson correlation method or Spearman Rank correlation depending of the parameter distribution. We used two-way ANOVA to compare morphologic and functional vestibular associations between scoliosis and control groups.

All analyses were carried out with SPSS software (IBM SPSS statistic 22.0), and *p*-values<0.05 were considered statistically significant.

## Results

### Group characteristics

Eighteen adolescents with AIS were included; one was considered as an outlier for morphologic vestibular parameters and was excluded from the study.

Further analyses were thus realized with a scoliosis group including 17 patients, and a control group including 9 participants. Both groups were similar in age and sex ratio and were free from otologic disease according to the ENT specialist (**Table 1**).

**Table 1:** Group characteristics.

	Scoliosis	Control	p-value
**Number**	17	9	
**Sex Ratio (M/F)**	4/13	3/6	0.60
**Age +/- SD (year)**	15.47 (+/- 1.84)	16.7 (+/- 1.5)	0.28
**Cobb angle (°) mean +/- SD [min-max]**	26.7 +/-8.3 [15–40]	NA	
**Dorsal scoliosis**	8	NA	
**Lumbar scoliosis**	5	NA	
**Thoracolumbar scoliosis**	4	NA	
**Right scoliosis**	8	NA	
**Left scoliosis**	6	NA	
**Right and left**	3	NA	

### Orientation of the lateral SCC: The left lateral SCC is more vertical in scoliosis

**Fig 4:**
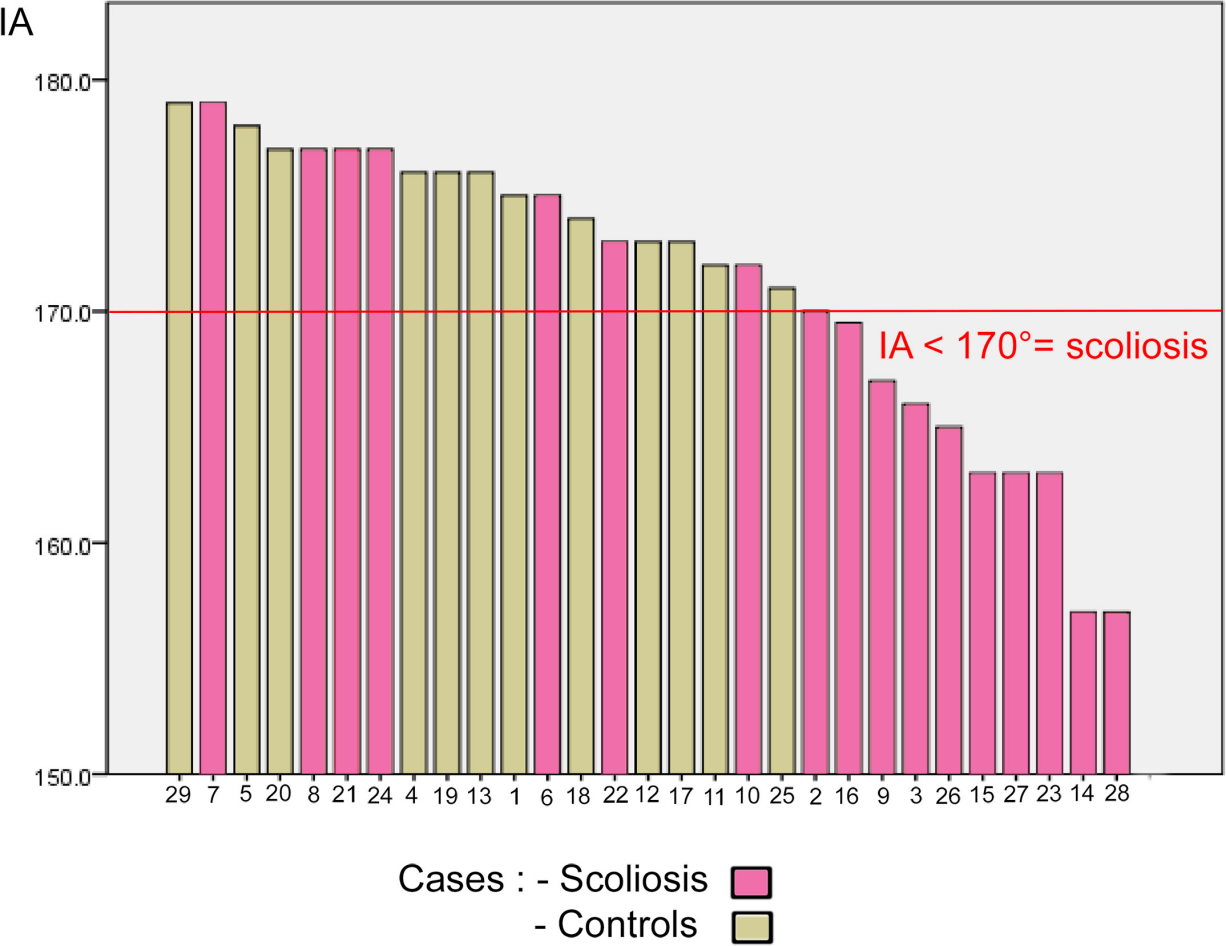
Intercanal Angle (IA) in scoliosis and controls. An IA less than or equal to 170° is 100% specific for scoliosis.

Both lateral SCCs formed a smaller angle together in the scoliosis group as measured by the IA (*p* = 0.004) with a large difference compared to control (*r* = 0.46) (**Table 2**). Consequently, an IA of less than or equal to 170° was 100% specific for scoliosis, with a sensitivity of 59% (**Fig 4**).

**Table 2:** Left lateral SCC is more vertical in scoliosis.

Orientation of Lateral Semicircular Canal	Scoliosis(1)	Control (1)	Significance(p)	t(df) or U (2)	Effect size « r »
**AI**	168.9 + /-7.0	175.1+ /-2.8	0.004**	t(23) = -3.25	0.46
**VLFR**	85+ /-5.4	85.3+ /-3.9	0.86	t(22) = -0.18	0.03
**VLFL**	83.9+ /-3.5	88.6+ /-1.9	<0.001**	t(23) = -4.4	0.60
**Diff VLFR-VLFL**	1.15+ /-6.0	-2.3+ /-3.3	0.15	t(23) = 1.5	0.30
**AbsolDiffVLFR-VLFL**	3.0 (13.6)	3.0 (11.8)	0.57	U = 58.0	0.11
**VLSR**	78.4+ /-6.6	77.7+ /-6.7	0.81	t(22) = -0.24	0.06
**VLSL**	79.0+ /-7.4	76.7+ /-5.3	0.47	t(21) = -0.73	0.16

(1) Mean (in degrees) +/- SD if T Test; or Median (in mm) (Mean Rank) if Mann Whitney U test.

(2) t value and degrees of freedom (df) if T Test; or U value if Mann Whitney U test.

**Significance < 0.01.

The difference in orientation mostly concerned the left lateral SCC, which is closer to vertical as illustrated by a much smaller VLFL (*p*<0.001; *r* = 0.6).

This vertical orientation of the left lateral SCC is strongly correlated with the position of the vestibule in the scoliosis group (*r* = -0.6; *p* = 0.02). The more vertical the left SCC orientation is (i.e. smaller VLFL), the further the left vestibule is from the midline (i.e. longer LDL, ADL, PDL).

Conversely, the right lateral SCC showed no significant difference between the two groups. This asymmetry between VLFR and VLFL was mostly left sided (58%) in the scoliosis group (VLFR-VLFL>0), but right sided (75%) in the control group (VLFR-VLFL<0) (*p* = 0.15).

Contrary to the frontal plane, analysis in the parasagittal plane showed no significant differences between the two groups **(Table 2**).

### Position of the SCC: the left canals are further from the midline in scoliosis

The left lateral SCC and the left posterior SCC were significantly further from the midline in scoliosis compared to controls. The left anterior SCC was also further from the midline in scoliosis, but the difference compared to controls was smaller and non significant (**Table 3**). Of note, the position of the left lateral SCC is strongly correlated with the vertical orientation of this canal (i.e VLFL). The further the canal was from the midline, the more vertical it became **(Table 4**). However, the right side exhibited neither significant differences between scoliosis and control (*r*<0.11, *p*>0.63) nor correlation between SCC position and lateral canal orientation **(Tables 3 and 4**).

**Table 3:** Left lateral SCC and posterior SCC are further from the midline in scoliosis.

Distances of Semicircular Canal vertex from the Midline	Scoliosis (1)	Control (1)	Significance(p)	T(df) or U (2)	Effect size « r »
**LDR**	42.9 + /-1.8	42.6+ /-1.3	0.63	t(20) = -0.49	0.11
**LDL**	43.9+ /-1.8	42.3+ /-3.9	0.01*	t(20) = -2.16	0.44
**Diff LDR-LDL**	-0.93+ /-1.5	0.29+ /-1.1	0.08	t(20) = -1.88	0.39
**[Diff LDR-LDL]**	1.0 (12.3)	1.0 (9.9)	0.40	U = 41.0	0.18
**ADR**	39.0+ /-0.5	40.0+ /-1.5	0.97	U = 52.0	0.01
**ADL**	40.2+ /-2.0	39.3+ /-1.1	0.19	t(20) = 1.12	0.24
**Diff ADR-ADL**	-0.13+ /-1.4	0.42+ /-3.3	0.43	t(20) = -0.80	0.18
**[Diff ADR-ADL]**	1.0 (10.6)	2.0 (13.4)	0.30	U = 39.0	0.22
**PDR**	40.4+ /-1.7	40.2+ /-1.6	0.74	t(20) = 0.30	0.08
**PDL**	41.0 (13.3)	40.0 (7.7)	0.05*	U = 26.0	0.41
**Diff PDR-PDL**	-0.8+ /-1.6	0.29+ /-1.7	0.18	t(20) = -1.40	0.30
**[Diff PDR-PDL]**	1.0 (11.7)	2.0 (11.1)	0.82	U = 49.0	0.05

(1) Mean (in mm) +/- SD if T Test; or Median (in mm) (Mean Rank) if Mann Whitney U test.

(2) t value and degrees of freedom (df) if T Test; or U value if Mann Whitney U test.

*significant results with *p*-value < 0.05.

**Table 4:** In scoliosis the orientation of the left lateral SCC is correlated with its position and other left SCC positions.

Parameter1	Parameter2	Coefficient of correlation	p-value
**VLFL**	LDL	-0.60	0.02*
	PDL	-0.60	0.02*
	ADL	-0.50	0.04*
**VLFR**	LDR	-0.12	0.67
	PDR	0.22	0.44
	ADR	0.25	0.37
**IA**	LDL	-0.26	0.35
	LDR	-0.38	0.16

*Significant results with *p*-value < 0.05.

### Function of the lateral SCC

Two adolescents with scoliosis exhibited a spontaneous nystagmus. The first was a permanent horizontal left nystagmus associated with a dorsal right-lumbar left scoliosis. This patient was excluded from the caloric test analysis because the spontaneous and caloric nystagmus were difficult to separate. The second patient exhibited an intermittent positional right nystagmus associated with an abnormal right canal paresis (35%) and was included in the group analysis. In the scoliosis group, vestibular excitability was lower but the difference was small (*r* = 0.238) and non significant (*p* = 0.25) compared to controls. The directional preponderance was abnormal (<2°/s) in half of the participants in both groups (*p* = 0.41).

Canal paresis was higher in the scoliosis group (mean = 18.25% +/- 16.37) compared to controls (mean = 10.33% +/- 5.87), but the difference was not significant according to the Mann Whitney test (*p* = 0.52). Six of the 16 adolescents with scoliosis had abnormal (>15%) canal paresis compared to only 1 in the 9 controls (X(1) = 1.99; *p* = 0.158) (**Table 5).**

**Table 5:** Lateral SCC function.

Caloric	Scoliosis	Control	Significance(p)	T(df) or U (1)	Effect size « r »
**Vestibular excitability (°/s)**	33.95+/-15.6	41.77+/-15.4	0.25	T(23) = -1.17	0.238
**Directional Preponderance(°/s)**	1.953+/-1.8	2.027+/-2.03	0.26	T(23) = -1.16	0.235
**Directional preponderance >2°/s (n/total)**	8/15	4/8	0.445		
**Canal paresis mean(%)**	18.25 +/-4.09	11.6 +/-1.67			
**Canal paresis median (%)**	13.2	11.2	0.52	U = 53.5	0.13
**Canal paresis >15% (n/total)**	6/16	1/9	0.218		

(1) t value and degrees of freedom (df) if T Test; or U value if Mann Whitney U test; or χ value (degrees of freedom) if Chi square test.

### Analysis within the scoliosis group

#### Analysis of location of the scoliosis

The IA was significantly different for the location of the scoliosis group. In particular, thoracolumbar scoliosis (*n* = 4) showed a smaller IA with a mean difference of 11.5° scoliosis compared to lumbar scoliosis (*n* = 5) (*p* = 0.033).

Thoracolumbar scoliosis also showed a more marked asymmetry in the location of lateral SCCs with a right canal located more laterally than the left compare with dorsal scoliosis (*p*<0.05) or lumbar scoliosis (*p*<0.03)

Two-way ANOVA combining location and side of scoliosis showed non significant main effects in morphologic or functional vestibular parameters.

#### Analysis of side of the scoliosis

Right and left scoliosis were comparable for morphologic and functional vestibular parameters (*p*>0.05).

We found no significant association between the side of scoliosis and the side of asymmetries for the position, orientation, or function of the lateral SCC.

#### Correlation of the Cobb angle with morphologic or functional vestibular parameters

No significant correlation was found between the Cobb angle and any morphological or functional parameters of the lateral SCC. (*p*>0.05).

We found no correlation between age and the Cobb angle nor with age and morphologic parameters of the lateral SCC.

### Relation between vestibular morphology and vestibular function

Scoliosis and control participants with abnormal canal paresis (i.e.>15°) exhibited more vertical lateral SCC as shown by the smaller IA, VLFR, and VLFL (*r*>0.4, *p*<0.05) (**Table 6**). Canal paresis (for both groups) correlated with the IA, the VLFR, the VLFL, the LDL, and the ADL (**Table 7**). Nevertheless, no significant difference was demonstrated for these parameters linked to the scoliosis group (*p*>0.75) or the side of abnormal canal paresis (*p*>0.056) (two-way ANOVA).

**Table 6:** Abnormal canal paresis is associated with more vertical lateral SCC.

Vestibular Morphology	Paresis <15% (1)	Paresis >15% (1)	Significance (p-value)	T(df) (2)	Effect size « r »
**IA**	173.06+/-5.0	165.57+/-7.8	0.01*	T(22) = 2.83	0.52
**VLFR**	86.35+/-4.1	82.29+/-4.7	0.046*	T(22) = 2.11	0.41
**VLFL**	86.71+/-3.1	83.29+/-3.5	0.026*	T(22) = 2.39	0.45

(1) Mean +/- SD in Scoliosis and control groups.

(2) t value (degrees of freedom) for T Test.

*Significant results with *p*-value < 0.05.

**Table 7:** Scoliosis and controls: Canal paresis is correlated with lateral SCC morphology.

Canal paresis correlation with	Coefficient correlation r	Significance p-value
**AI**	-0.60	0.001**
**VLFR**	-0.433	0.02*
**VLFL**	-0.422	0.04*
**VLSR**	0.193	0.38
**VLSL**	0.22	0.33
**LDR**	0.343	0.1
**LDL**	0.42	0.04*
**PDR**	0.178	0.40
**PDL**	0.244	0.25
**ADR**	0.002	0.99
**ADL**	0.507	0.01*

Pearson correlation for parametric distribution, or Spearman Rank.

*Significant results with *p*-value < 0.05.

** Significant results with *p*-value<0.01.

## Discussion

This study shows that adolescents with idiopathic scoliosis present an inner ear asymmetry characterized by a left semicircular canal which is more vertically oriented compared to a control group. This view is in accordance with the results of Shi et al. who demonstrated, in girls with left scoliosis, a shorter distance between the centers of the lateral SCC and the superior SCC on the left side [52]. Our study includes right and left scoliosis with no significant differences between both sides, but as in Shi et al. study the labyrinth abnormality was only observed in the left side [52]. The concordant abnormality described by Shi et al. and in our study implies a malformation of the labyrinth itself. Additionally, our study showed that the left labyrinth in adolescents with scoliosis presents a lateral and a posterior canal located more laterally compared to a control group. This could result from a malformation either of the labyrinth itself, or of the entire petrous bone. Enlarged left skull vaults have been seen in scoliosis [10], but no information about the petrous bone is available at the present time. Of note, the orientation of the lateral SCC is strongly correlated with the position of the 3 SCCs, indicating that both are due to a labyrinth malformation or that the left labyrinth malformation is associated with a malformed left petrous bone.

In either case, these asymmetries of the labyrinth and the skull complement other published findings of asymmetries in the scoliotic skeleton including the pelvis, the mandible, and the ribs [7–10]. These asymmetries support a biomechanical theory whereby one anomaly could mechanically provoke other deformities[10,27,53–56] [10,27,53–55]. However, no link between these asymmetries is demonstrated and they could be independent or due to a common cause.

**Fig 5:**
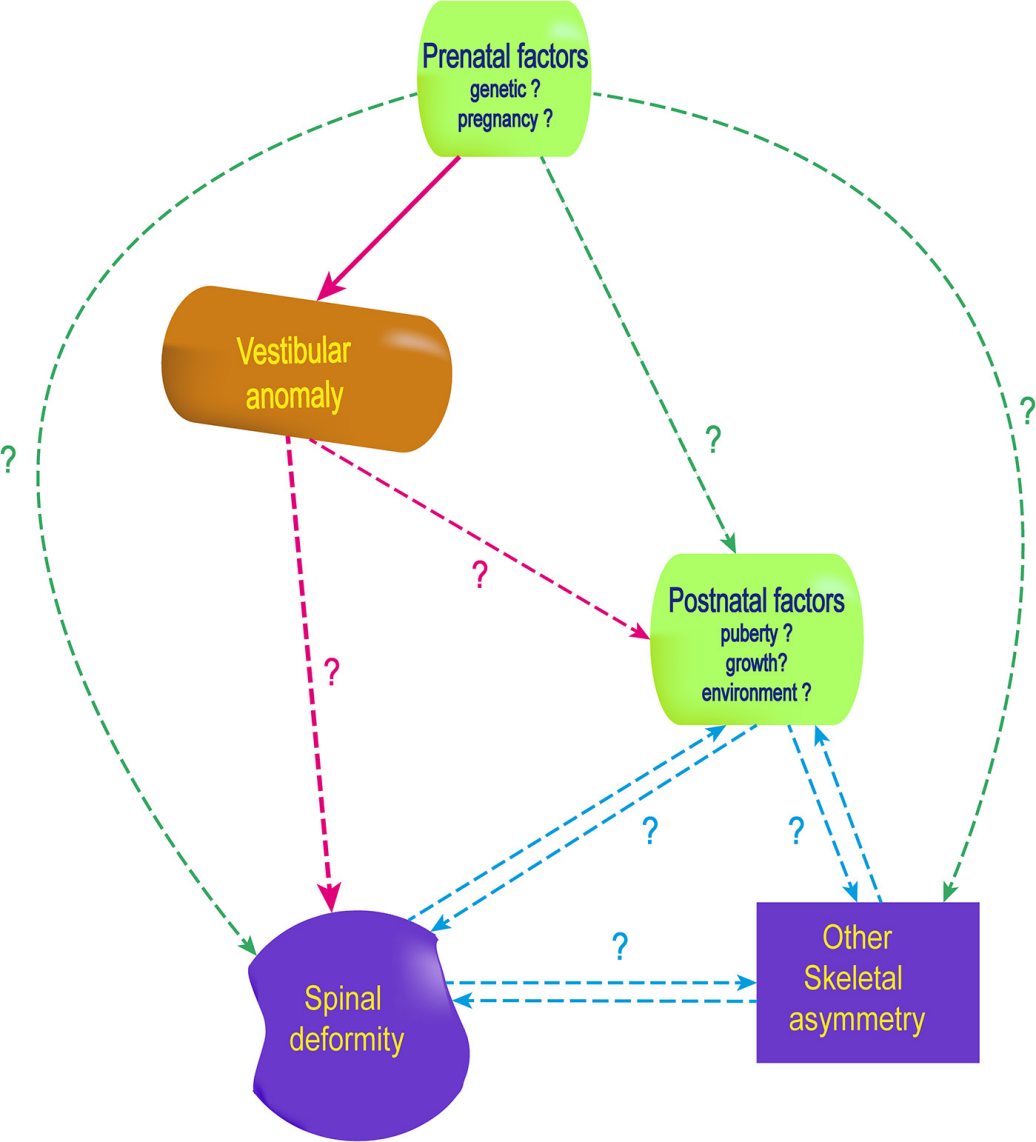
Early anomalies of the vestibular system in AIS pathogenesis and consequences in adolescent idiopathic scolisosis pathogenesis. The pink dash arrows represent the hypothesis that a vestibular anomaly may be the cause of later anomalies, including spinal deformity. Different mechanisms supporting this hypothesis are illustrated in Fig 6.

According to Cox et al., the orientation of the lateral SCC (related to midsagittal or horizontal planes) does not change during embryogenesis [57], and the shape and orientation of the labyrinth is normally fixed early because of prenatal ossification [36–43]. This particularity of the bone of the labyrinth is explained by a high expression of *opg* gene, associated with a low expression of *bmp3* gene [58] responsible for an inhibition of bone remodeling and resulting in the densest bone in the human body [38,59–63]. Consequently, anomalies observed in an adolescent’s labyrinth, would already have been present at birth and probably before the 35^th^ week *in utero*. Thus, the labyrinth asymmetry would not be the consequence of the spine deformity but rather its cause. If this is not the case, both abnormalities would come from a common cause or each from a separate origin (**Fig 5**).

Among these three hypotheses, animal models argue for a vestibular impairment causing the spine deformity. Indeed experimental vestibular lesion in animals induces scoliosis[34,35]. Studies in Xenopus underline that vestibular impairment needs to be early (before metamorphosis) to induce osseous deformation, which is in accordance with our hypothesis of early bony labyrinth anomalies in AIS [35].

Our study found abnormal canal paresis in more than one-third of the AIS group. Results of functional tests show substantial variation within the scoliosis group which may decrease the statistical significance when compared to the control group. We noted a correlation between a deficit in the caloric test and the angle of the lateral SCC in the frontal plane (i.e. IA, VLFL, and VLFR). This could suggest that the verticalization of the lateral SCC either reduces the conduction of the water temperature during the test, or is associated with a functional impairement.

In a previous study with caloric testing in AIS, Sahlstrand and al. found a significantly lower vestibular response located on the side of the spine concavity in AIS [33]. But they found no significant difference in a control group [33], and neither did Wiener et al.[14]. Additionally, Sahlstrand and al. observed occulomotor anomalies in more than half of their AIS group that were either spontaneous (15%) or positional (36%) nystagmus (without describing the orientation). Spontaneous nystagmus can signify a SCC impairment, and in our study the two horizontal nystagmus more specifically indicated a lateral SCC. Positional nystagmus is interpreted by Sahlstrand et al. to originate from the brainstem [33].

In addition to the lateral SCC, the otolith function could also be impaired in AIS since the utricle is known to be roughly parallel to the lateral SCC [64]. The function of otolith sensors (i.e. utricle and saccule) have been studied using off-vertical-axis rotation in patients with scoliosis. Sixty-seven percent exhibited a significantly greater value of directional preponderance, arguing for an impairment of the vestibulospinal function [14].

Additionally, AIS patient show worse sway (relative to controls) when vestibular inputs are the only available [15]. Taken together these studies suggest a vestibular system involved in AIS. Yet anomalies of the vestibular organ alone are not sufficient to explain long-term postural impairment leading to spinal deformity.

Complete vestibular asymmetry (e.g. unilateral labyrinthectomy or vestibular neurectomy) results in postural symptoms which quickly disappear due to central compensation [65,66]. In addition, in normal human, the brain manages physiological asymmetry because of vestibular predominance in the side of handedness [67,68].

**Fig 6:**
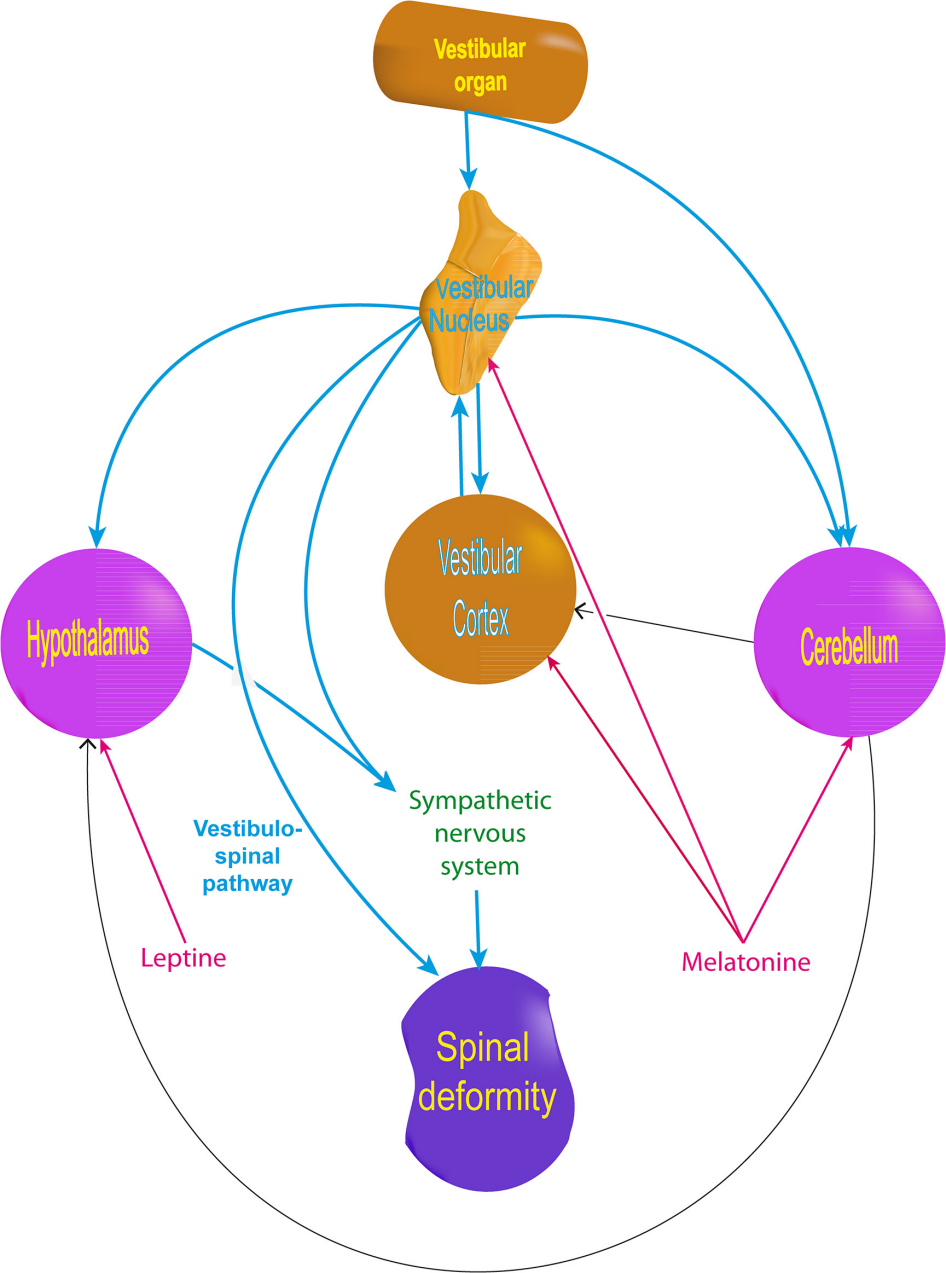
Influence of the vestibular system on key elements involved in AIS pathogenesis theories, including hormonal and neurosensory theories.

AIS symptoms could thus result from vestibular organ abnormalities associated with brain impairments which can be detected by MRI studies. White matter in the corpus callosum and left corticothalamic tract shows changes [30]. Cortical areas, including vestibular cortices, are thinner and there are changes in the cortical network [31,69,70]. The ventral brainstem is also asymmetric and the cerebellum is thicker in the right lobules VIIIa, VIIIb, and the bilateral lobules X [32,71].Of note, the cerebellum (including lobule VIII) is connected to the vestibular system either directly from the vestibular organ or through the vestibular nuclei complex [72–78] (**Fig 6**). Moreover, the cerebellum is particularly rich in melatonin receptor, a hormone suspected of acting in scoliosis pathogenesis [12,55,79]. Melatonin reciprocally interacts with the vestibular system by influencing the firing of the medial vestibular nuclei [80], the vestibulosympathetic system [81], and balance [82] (**Fig 6**).

Anomalies of the central nervous system in AIS may constitute an adaptation to compensate for the instability induced by the spine deformity [32]. They also could result from early abnormal vestibular input occurring at a critical period for the central nervous system. The lack of appropriate inputs during critical periods induce permanent brain abnormalities, as demonstrated for the visual [83], auditory [84] and tactile [85] sensory systems. Critical periods are demonstrated for the vestibular system [86,87] and for multisensory integration as well. Therefore, brain anomalies and multisensory impairments demonstrated in AIS could result from early vestibular abnormalities appearing before the critical period [15,16,88,89]. This point is reinforced by animal models where scoliosis appears only if vestibular lesion is early enough [35].

Finally, in addition to early modification of the central nervous system and the vestibulospinal pathway, the vestibular system could also contribute to spine deformity by influencing the neuroendocrine or the vestibulosympathetic system (**Fig 6**). In fact, the lateral vestibular nuclei and the vestibular cortex (e.g. the insula) project out to the lateral hypothalamus which is rich in leptin receptors [90–93]. Leptin is one of the main hormones suspected of being involved in scoliosis [27]. The lateral hypothalamus, the cerebellum, and the rostro-ventro-lateral medullary structur also influence the vestibulosympathetic system [94–96] which regulates bone remodeling [97,98]. Therefore, the vestibular sympathetic system may contribute to bone deformity and to the bone demineralization demonstrated in adolescent scoliosis [99–102].

Altogether, these results are in accordance with: 1) in the biomechanical theory of AIS, early asymmetry exists before the spine deformity and may therefore be its cause rather than its consequence; 2) in the endocrine theory, the vestibular anomalies could interact with hormones early before puberty; 3) in the neurosensory theory, vestibular impairment may start early enough to influence brain maturation and might precede the biomechanical or hormonal phenomena.

The multiple consequences of an early vestibular impairment, including some compensatory mechanisms, may explain why the lateral SCC parameters are not directly correlated with the Cobb angle. Another reason may be that scoliosis is a dynamic process and etiologic factor(s) may correlate to the progression of the process rather than the degree of spine deformity in one measurement. Furthermore, the mild spine deformities of our participants (Cobb’s angle < 40°) may decrease the chance of demonstrating statistical correlation. The influence of these last two factors was demonstrated in sway in AIS where participants exhibit more impairment in case of deformities > 40° or progression > 10°/ year. [103]

Additionally, other factors (e.g. sex, body mass index, internal organ asymmetry) may also explain the lack of correlation, for example between the left lateral SCC anomalies and the side of the scoliosis [27,104–107]. The involvement of late factors appearing during childhood or puberty is also likely to explain why the spine deformity appears only in adolescence despite the early vestibular anomaly. Lastly, vestibular anomaly may also act as an onset factor of scoliosis without further role in progression.[108]

Finally our results lead to another question: What is the origin of the lateral SCC anomaly? The estimate of early occurrence of the malformation focuses the answer on genetic factors and/or environmental factors during pregnancy. This underscores that lateral SCC malformation and spine deformity could also be independent from each other but induced by a common cause. One hypothesis of common causes could be genes involved in growth acceleration occurring during SCC embryogenesis [42] and puberty.

## Conclusion

We have used the labyrinth of adolescents as a “living fossil” to explore the chronology of AIS and to show that anomalies of the vestibular system start before birth. As the earliest anomaly demonstrated so far, the vestibule may be the cause of the spinal deformity. If not, both anomalies might still result from a common cause which appears before birth according to the timing of the vestibular malformation. In either case, findings encourage the search during pregnancy for causative environmental factors which could lead to prenatal preventive treatment. Moreover, a simple MRI measurement of the lateral SCC, as demonstrated here, could be used to predict AIS and initiate preventive treatment during childhood. However, the best treatments will certainly need a complete understanding of AIS chronology which requires multiple landmarks. The lateral semicircular canal constitutes a first milestone which can now open the road to longitudinal studies.

## Supporting Information

S1 Table Data set of each participant. (XLSX)
